# Association between 24-h movement guidelines and cardiometabolic health in Chilean adults

**DOI:** 10.1038/s41598-022-09729-1

**Published:** 2022-04-06

**Authors:** Ricardo Riquelme, Leandro F. M. Rezende, Adilson Marques, Clemens Drenowatz, Gerson Ferrari

**Affiliations:** 1 Facultad Medicina, Escuela de Nutrición y Dietética, Universidad de los Andes, Santiago, Chile; 2 Department of Preventive Medicine, Escola Paulista de Medicina, Universidade Federal de São Paulo, Sao Paulo, Brazil; 3 CIPER, Faculdade de Motricidade Humana, Universidade de Lisboa, Lisbon, Portugal; 4 ISAMB, Universidade de Lisboa, Lisbon, Portugal; 5 Division of Sport, Physical Activity and Health, University of Education Upper Austria, Linz, Austria; 6 Universidad de Santiago de Chile (USACH), Escuela de Ciencias de la Actividad Física, el Deporte y la Salud, Santiago, Chile; 7 Laboratorio de Rendimiento Humano, Grupo de Estudio en Educación, Actividad Física y Salud (GEEAFyS), Universidad Católica del Maule, Talca, Chile

**Keywords:** Diseases, Risk factors, Disease prevention, Public health

## Abstract

This study aimed to examine the association between meeting 24-h movement guidelines and cardiometabolic health in Chilean adults. We used cross-sectional data of 2618 adults from the Chilean National Health Survey 2016–2017. Meeting the 24-h movement guidelines was defined as ≥ 600 MET-min/week of physical activity; ≤ 8 h/day of sitting time; and 7 to 9 h/day of sleep duration. Cardiometabolic health indicators were body mass index, waist circumference, high triglycerides, high blood pressure, type 2 diabetes, metabolic syndrome, and risk of cardiovascular disease in a 10-year period. Meeting none out of three 24-h movement guidelines (vs all three) was associated with higher odds of overweight/obesity (OR 1.67; 95%CI 1.45 to 1.89), high waist circumference (1.65; 1.40 to 1.90), hypertension (2.88; 2.23 to 3.53), type 2 diabetes (1.60; 1.26 to 1.94), metabolic syndrome (1.97; 1.54 to 2.40) and risk of cardiovascular disease (1.50; 1.20, 1.80). Meeting one guideline (vs three) was associated with higher odds of five of out seven cardiometabolic indicators. Our study found that the composition of movement behaviors within a 24-h period may have important implications for cardiometabolic health.

## Introduction

From a movement perspective, the 24-h period is distributed among physical activity of various intensities (light, moderate and vigorous), sedentary behaviors and sleep duration. Engaging in sufficient levels of physical activity, limiting sitting time, and adequate sleep duration throughout the day have been associated with several health benefits across the lifespan^1–4^. Traditionally, studies have focused on investigating independent associations of physical activity, sitting time, and sleep duration with different health outcomes, or with only partial adjustment for time spent in other movement behaviors^5^. However, because recent studies revealed that these movement behaviors may interact with each other^5^, there is growing interest in an integrated approach to movement behavior studies. Previous studies have showed the independent and joint association of physical activity, sitting time, and sleep duration with poor cardiometabolic health indicators (i.e., adiposity level, HDL-cholesterol, and triglycerides)^6–8^.

The world’s first 24-h movement guidelines that integrate physical activity, sitting time, and sleep duration was published by Canada^7,9^, and soon after by Finland, New Zealand, Australia, and in some other countries^10–13^. As part of such efforts, the World Health Organization (WHO)^14^ and the National Sleep Foundation^15^ offer recommendations for physical activity and sleep duration for different age groups. Although no specific benchmark is available for sitting time, it is generally recommended to minimize time spent in sedentary behaviors, mainly sitting time^16,17^. To date, no evidence is available to understand the levels of movement behaviors in Latin American countries inhabitants based on internationally recognized benchmarks such as global physical activity guidelines or international sleep duration recommendations^14,15^. The guidelines represent a new approach to health promotion by including several general recommendations over a that include time spent in physical activity, sitting time, and sleep duration^7^.

Chile, a high-income Latin American country, has experienced a rapid epidemiological and nutritional transition^18^. Of note, the prevalence of overweight and metabolic syndrome (defined as a cluster of risk factors that include abdominal obesity, hypertension, hyperglycemia, and dyslipidemia) reached 78% and 13%, respectively^19,20^. Furthermore, the prevalence of type 2 diabetes mellitus increased from 4.2% in 2003 to 12.3% in 2016^19^. These transitions might be partially explained by insufficient levels of physical activity, high sitting time, and inadequate sleep duration throughout the day. However, to our knowledge, no studies in Chile and Latin American region have examined the association of meeting 24-h movement guidelines with cardiometabolic health indicators.

In this study, we estimated the prevalence of meeting the general and specific combinations of 24-h movement guidelines by sociodemographic characteristics in Chilean adults. We also examined the associations of meeting general and specific-combinations of 24-h movement guidelines with cardiometabolic health indicators.

## Material and methods

### Study design and sample

We obtained data from the National Health Survey of Chile (NHS) 2016–2017^19^. The NHS 2016–2017 was a cross-sectional, household survey that enrolled 6233 participants aged 15 years and older, who habitually reside in private homes located in urban and rural areas of the fifteen regions of Chile^19^. A complex, multistage sampling strategy was performed, considering counties as the primary sampling unit, households as the secondary sampling unit, and one participant from selected households as the tertiary sampling unit. Sampling weights from the survey accounted for differences in selection probability and non-response rates^19^. The post-stratification adjustment allowed to expand the sample to the estimated population in Chile. Data collection was carried out between August 2016 and March 2017. One participant per household was randomly selected using a Kish computational algorithm, and the response rate was 67%. Details on NHS 2016–2017 are available elsewhere^19^.

**Figure 1:**
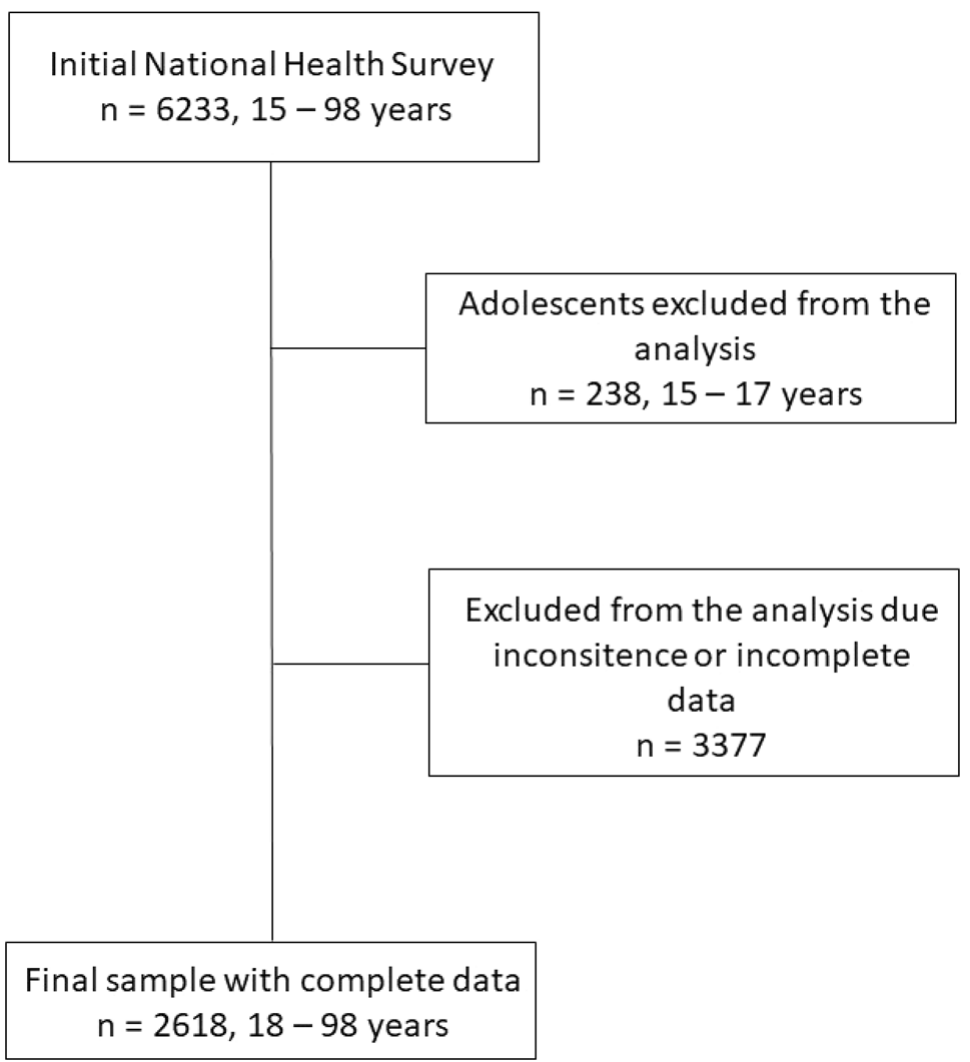
Flow chart of the process to obtain the final sample.

We excluded from our study adolescents aged 15–17 years (n = 238) and participants with missing or incomplete data of physical activity, sitting, sleep duration, or cardiometabolic health (n = 3377). Thus, our final analitical sample included 2618 adults (Fig. 1).

### Assessing the 24-h movement guidelines

The Global Physical Activity Questionnaire (GPAQ) was used to assess physical activity and sitting time^21,22^. Developed by the WHO to measure population-level physical activity behaviors, the GPAQ uses standardized protocols and have shown to be a valid and reliable instrument to incorporate cultural and other differences^21,22^. Participants provided information on the duration, frequency, and intensity of physical activities performed in three domains (occupational, active commuting, and recreational). For each domain, metabolic-equivalent tasks (MET; where 1 MET =  ~ 3.5 ml O^2^ kg^−1^ Min^−1^) were assigned according to the GPAQ protocol (4-METs was used for moderate and transport-related activities and 8-METs for vigorous activities). Total self-reported physical activity was calculated as the sum of MET-min/week^−1^ across all three domains. Participants were subsequently categorized as physically inactive (< 600 MET-min/week^−1^) or active (≥ 600 MET-min/week^−1^)^23^. Prevalences were based on 600 MET-min/week, which is approximately 150 min/week of moderate to vigorous physical activity^24^.

A single question from the GPAQ was used to measure sitting time^25,26^. The question was (i) “How much time do you usually spend sitting or reclining at work, at home, getting to and from places, or with friends including time spent sitting at a desk, sitting with friends, travelling in car, bus, or train, reading, playing cards or watching television, but do not include time spent sleeping on a typical day?” The participant responded in hours and minutes per day. This question has shown fair validity as was similarly reported in other countries (r = 0.23 to 0.26)^25,26^. We adopted the cutpoint of spend ≤ 8 h/day as guideline for sitting time^7,9^.

Self-reported sleep duration was assessed using two items, which asked participants to report their time spent sleeping on a typical day^19^. The sleep questionnaire consisted of two questions examining the sleep duration (hours/day): “How much time did you usually sleep on weekdays and weekends?” These questions were asked separately for weekdays and weekend days. The average sleep duration per day was calculated as follows = [(weekday time*5) + (weekend day time*2)]/7^19^. Sleep duration values were dichotomized into meeting (≥ 7 and ≤ 9 h/day) or not meeting (< 7 or > 9 h/day) the sleep duration guidelines^7,9^.

### Cardiometabolic health indicators

All measurements of cardiometabolic health were taken by previously trained professionals, using standard protocols^19^. The collected variables were body height and weight, waist circumference, triglycerides, blood pressure, and indicators for type 2 diabetes. In addition, based on the abovementioned cardiometabolic indicators, metabolic syndrome status and risk of cardiovascular disease were determined^19^.

Height was measured with a portable stadiometer with accuracy to the nearest 0.1 cm)^19^. Weight was measured with a digital scale (Tanita HD713) with an accuracy of 0.1 kg^19^. Weight measurements were taken barefoot, and the participants wearing light clothing. Body mass index (kg/m^2^) was calculated (weight [kg]/height [m^2^]) and participants were categorized as underweight/eutrophic (≤ 24.9 kg/m^2^) or overweight/obesity (≥ 25.0 kg/m^2^)^19,27^.

Waist circumference (cm) was measured midpoint between the lower coastal ridge and the upper margin of the superior iliac crest, using a flexible plastic tape^19^. Central obesity was defined as > 88 cm for women and > 102 cm for men^19,28^.

Venous blood samples were obtained after at least 8 h of fasting according standardized methods that have been described previously^19^. Participants with circulating triglycerides ≥ 150 mg/dL; HDL-C < 40 mg/dL for men, or < 50 mg/dL for women, or under drug treatment for cholesterol control were considered as high triglycerides^19^.

Blood pressure was measured with an Omron HEM-7200 Monitor and participants being seated^19,29^. Hypertension was defined by a measured systolic blood pressure ≥ 140 mmHg or diastolic blood pressure ≥ 90 mmHg or self- reported antihypertensive treatment^19^.

Type 2 diabetes was determined by the presence of any of the following three criteria: (a) self-reported medical diagnosis of type 2 diabetes, (b) being under medical prescription for type 2 diabetes, (c) having fasting baseline glycaemic values > 126 mg/dL^19^.

Metabolic syndrome was defined according to the Chilean National Guidelines as having at least three of the following five components: high waist circumference (> 90 cm for men and > 80 cm for women), low HDL, hypertension (HDL-C 130/85 mmHG or under BP-lowering treatment) and impaired fasting glucose (IFG, glucose > 5.6 mmol/L or under treatment with antidiabetic drugs)^19^.

The risk of cardiovascular disease was evaluated by the Framingham Risk Score^30^, which was adapted for a Chilean population^19,31,32^. The Framingham Risk Score estimates the possibility of suffering a cardiovascular event or stroke in a 10-year period for people without a history of a previous cardiovascular event^31^. Participants were classified as low (< 5%) and middle/high (≥ 5%) risk of cardiovascular disease in a 10-year period^19,31,32^.

### Sociodemographic correlates

Sociodemographic characteristics included sex (women/men), age (adults [18–64 years], and older adults [≥ 65 years])^7^, education level (up to primary [< 8 years of studies], secondary [between 8 and 12 years of studies] and beyond secondary [> 12 years of study]), monthly household income (stratified into tertiles: lowest [< US $310.00], medium [US $310.00–705.00], and highest [> US 705.00]), health insurance (private [Isapres], public [Fonasa] or other/none), and indigenous ethnicity (yes/no)^19^. In Chile, there are two main ethnicities; the first relates to Indigenous, and the second relates to those with other roots^33^. We also considered urban–rural geographic areas based on the Chilean population census^19^. Lifestyle risk factors included low consumption of fruits and vegetables (≤ 4 days per week)^20^, tobacco smoking (never/former or smoker)^20^, any alcohol consumption (using the short version of Alcohol Use Disorder Identifcation Test (AUDIT-C)^34^, adapted and validated for Chile residents^35^.

### Statistical analysis

Descriptive data were presented as means, standard deviation (SD), frequency, and proportions according to sociodemographic correlates and cardiometabolic health. Each participant was categorized as either “meeting” or “not meeting” the 24-h movement guidelines as follows: (1) engage in ≥ 600 MET-min/week of physical activity; (2) spend ≤ 8 h/day in sitting time; and (3) obtain between 7 and 9 h/day of sleep duration^7,9^. The number of 24-h movement guidelines (0–3) met was created. For instance, the participants who met all three recommendations for physical activity, sitting time and sleep duration were categorized as meeting the integrated all three movement guidelines. In the specific-combination of 24-h movement guidelines, the proportion of participants meeting the physical activity, sitting time, and sleep duration and combinations of the guidelines (“none”, “only physical activity guideline met”, “only sitting time guideline met”, “only sleep duration guideline met”, “both the physical activity and sitting time guidelines met”, “both the physical activity and sleep duration guidelines met”, “both the sitting time and sleep duration guidelines met”, and “all three guidelines met”) were also calculated.

Multivariable logistic regression models were performed to estimate odds ratios (OR) and 95% confidence interval for the association between 24-h movement guidelines and cardiometabolic health indicators (dependent variable). Models were adjusted for the following potential confounders: region, sex, age, education level, monthly household income, health insurance, ethnicity, geographic area, fruits and vegetables consumption, tobacco consumption, and alcohol consumption. All analyses considered the NHS 2016–2017 complext sampling design^19,20^. Weights took into account the complex sampling design and the four levels of multistage sampling. All statistical analyses were conducted using SPSS V28 software (SPSS Inc., IBM Corp., Armonk, New York, NY, USA). A significance level of p < 0.05 was adopted.

### Ethics approval and consent to participate

The NHS was funded by the Chilean Ministry of Health and approved by the Research Ethics Committee of the Faculty of Medicine of the Pontificia Universidad Católica de Chile (project number 16-019). Informed consent was obtained from all subjects and/or their legal guardian(s). All aspects of the study were in accordance with the Declaration of Helsinki and were performed in accordance with relevant guidelines and regulations.

## Results

Participants sociodemograhic characteristics, lifestyle risk factors and cardiometabolic health indicators by 24-h movement guideline groups are presented in Table 1. The total number of participants included in the study was 2618 (1661; 63.4% women) with a mean age of 49.4 years (SD: 18.9). Overall, 25.1% were older adults (≥ 65 years), 54.8% had 8 to 12 years of education, 49.7% were in the lowest of household income group, 88.2% had access to health insurance, 88.3% were not of indigenous heritage, 82.7% lived in urban areas, 50.9% ate ≤ 4 days/week of fruit and vegetables, 73.2% were never/former tobacco smokers and 73.9% consumed alcohol. Most participants (76.4%) were classified as living with overweight/obesity. Almost half (46.1%) had high waist circumference, 33.7% had high triglycerides, 36.6% had hypertension, 15.5% had type 2 diabetes, 45.1% had metabolic syndrome, and 30.9% had a high risk of cardiovascular disease (Table 1). We did not observed differences (p > 0.05) between the participants who had complete data and those who were excluded from the analytical sample in terms of sex, age group and educational level (data not shown).

**Table 1:** Participants socidemographic characteristics, lifestyle risk factors, and cardiometabolic health indicators according to 24-h movement guidelines.

	Total (n = 2618)	Met none guidelines (%)	Met one of the three guidelines (%)	Met two of the three guidelines (%)	Met all three guidelines (%)
**Sociodemografic correlates (%)**					
Categorical age					
Adults (18–64 years)	1961 (74.9)	74.9	80.5	77.3	69.0
Older adults (≥ 65 years)	657 (25.1)	25.1	19.5	22.7	31.0
Sex					
Men	957 (36.6)	53.3	44.8	39.1	29.0
Women	1661 (63.4)	46.7	55.2	60.9	71.0
Education level					
Up to primary (< 8 years)	649 (24.8)	26.7	20.7	25.1	26.3
Secondary (8–12 years)	1435 (54.8)	53.3	58.2	54.6	53.5
Beyond secondary (> 12 years)	534 (20.4)	20.0	21.1	20.3	20.2
Monthly household income					
Lowest	1302 (49.7)	46.7	48.6	49.4	50.8
Medium	939 (35.9)	46.6	37.6	36.0	34.7
Highest	377 (14.4)	6.7	13.8	14.6	14.5
Health insurance					
Private	212 (8.1)	13.3	8.9	7.6	8.3
Public	2308 (88.2)	80.0	87.6	88.2	88.4
Other/none	98 (3.7)	6.7	3.5	4.2	3.3
Indigenous ethnicity					
Yes	306 (11.7)	0.0	9.9	11.4	13.2
No	2312 (88.3)	100.0	90.1	88.6	86.8
Geographic area					
Urban	2164 (82.7)	86.7	85.2	82.7	81.4
Rural	454 (17.3)	13.3	14.8	17.3	18.6
Fruits and vegetables consumption					
≤ 4 days/week	1333 (50.9)	53.3	49.5	52.0	50.0
> 4 days/week	1285 (49.1)	46.7	50.5	48.0	50.0
Tobacco consumption					
Current	701 (26.8)	40.0	37.1	29.9	23.4
Never/former	1917 (73.2)	60.0	62.9	70.1	76.6
Alcohol consumption					
Yes	1934 (73.9)	54.5	74.0	74.9	72.7
No	684 (26.1)	45.5	26.0	25.1	27.3
**Cardiometabolic health**					
Body mass index (%)					
Underweight/eutrophic	619 (33.6)	20.0	25.1	24.7	21.6
Overweight/obesity	1999 (76.4)	80.0	74.9	75.3	78.4
Waist circumference					
Below threshold	1411 (53.9)	33.3	56.1	56.3	49.9
Above threshold	1207 (46.1)	66.7	43.9	43.7	50.1
Triglycerides					
Normal	1735 (66.3)	73.3	66.4	65.4	67.2
High	883 (33.7)	26.7	33.6	34.6	32.8
Hypertension					
No	1659 (63.4)	73.3	66.7	64.5	60.1
Yes	959 (36.6)	26.7	33.3	35.5	39.9
Type 2 diabetes					
No	2212 (84.5)	86.7	88.5	84.3	83.1
Yes	406 (15.5)	13.3	11.5	15.7	16.9
Metabolic syndrome					
No	1437 (54.9)	73.3	58.2	55.1	52.7
Yes	1181 (45.1)	26.7	41.8	44.9	47.3
Risk of CVD					
Low	1178 (45.0)	80.0	72.8	69.8	66.1
Middle/high	1440 (55.0)	20.0	27.2	30.2	33.9

*CVD* Cardiovascular disease.

Participants meeting all three 24-guideline recommendations were more likely older, women, had lower household income and higher acces to public health insurance, indigenous, and living in rural area compared to those meeting none of the guidelines. Participants meeting all three 24-guideline were also more likely never smokers and had higher consumption of fruits and vegetable and alcohol compared to those meeting none of the guidelines (Table 1).

The prevalences of meeting general and specific combinations of the 24 h movement guidelines are presented in Table 2. Overall, 18.1% (95% CI 15.5, 20.7) of the sample met all three recommendations, 44.7% (95% CI 41.5, 47.9) met two, 33.1% (95% CI 29.8, 36.4) one, and 4.1% (95% CI 2.5, 5.7) met none of the three recommendations. We also found the following prevalences of meeting the specific guidelines: 22.0% (95% CI 18.1, 25.9) for physical activity, 15.2% (95% CI 11.7, 18.7) for sitting time, 6.5% (95% CI 4.1, 8.9) for sleep duration. In addition, 12.6% (95% CI 9.9, 15.3) of participants met physical activity and sitting time, 9.5% (95% CI 8.9, 10.1) met sleep duration and sitting time, while 12.0% (95% CI 8.1, 15.9) of participants met physical activity and sleep duration guidelines (Table 2).

**Table 2:** Proportion [% (95% CI)] of participants meeting the physical activity, sitting time and sleep duration and combinations of these guidelines.

Meeting guidelines	Total (n = 2618)	
	n	% (95% CI)
**General combinations of movement behaviors, %**		
All three (physical activity and sitting time and sleep duration)	473	18.1 (15.5 to 20.7)
Two out of three	1171	44.7 (41.5 to 47.9)
One out of three	867	33.1 (29.8 to 36.4)
None	107	4.1 (2.5 to 5.7)
**Specific combinations of movement behaviors, %**		
All three (physical activity and sitting time and sleep duration)	473	18.1 (15.5 to 20.7)
Physical activity and sitting time	329	12.6 (9.9 to 15.3)
Physical activity and sleep duration	315	12.0 (8.1, 15.9)
Sleep duration and sitting time	249	9.5 (8.9 to 10.1)
Only physical activity	577	22.0 (18.1 to 25.9)
Only sitting time	397	15.2 (11.7 to 18.7)
Only sleep duration	171	6.5 (4.1 to 8.9)
None	107	4.1 (2.5 to 5.7)

Meeting guidelines was defined as ≥ 600 MET-min/week^−1^ of physical activity, ≤ 8 h/day of sitting time, and between 7 and 9 h/day of sleep duration.

**Figure 2:**
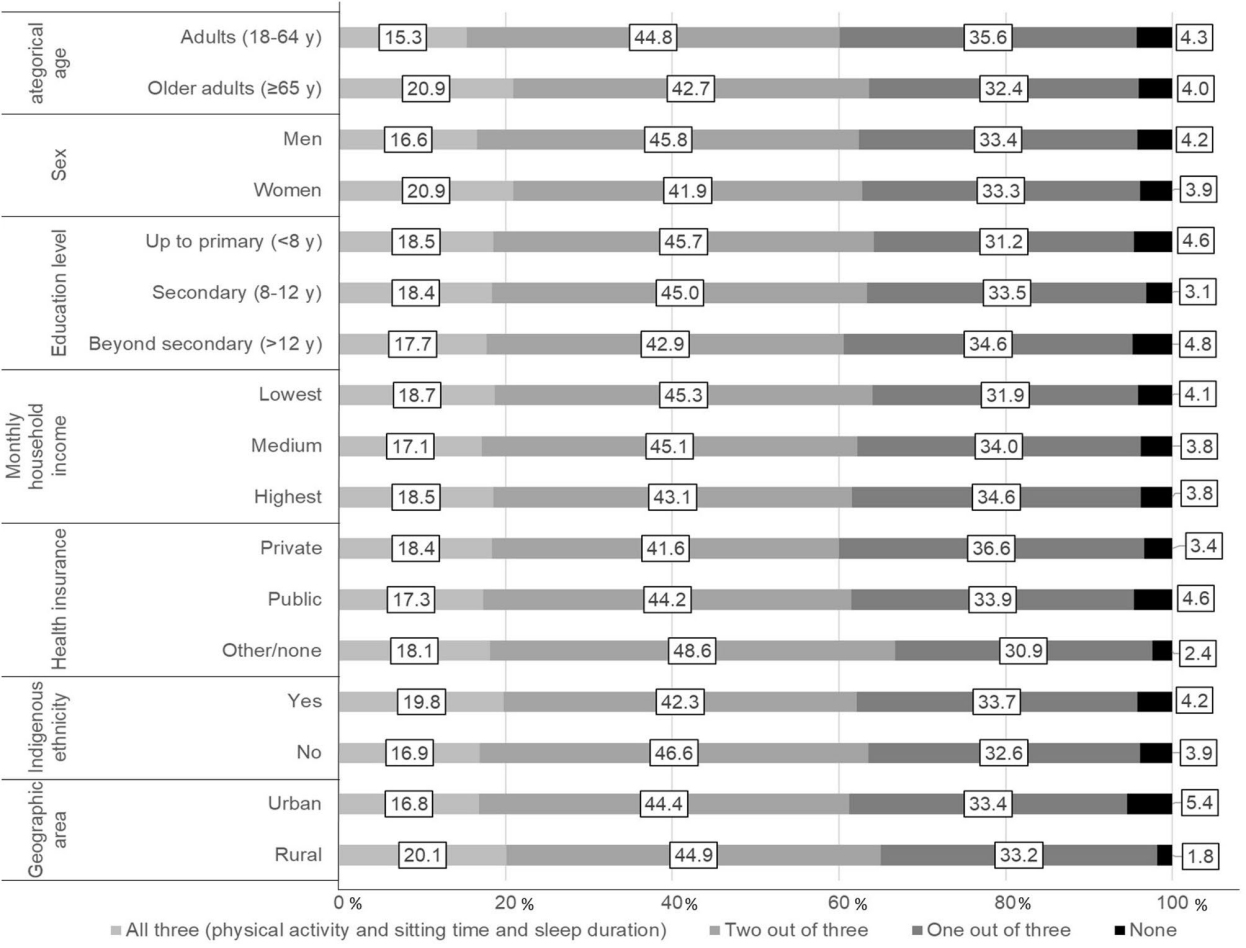
Proportion (%) of participants meeting the general combinations according sociodemographic characteristics.

**Figure 3:**
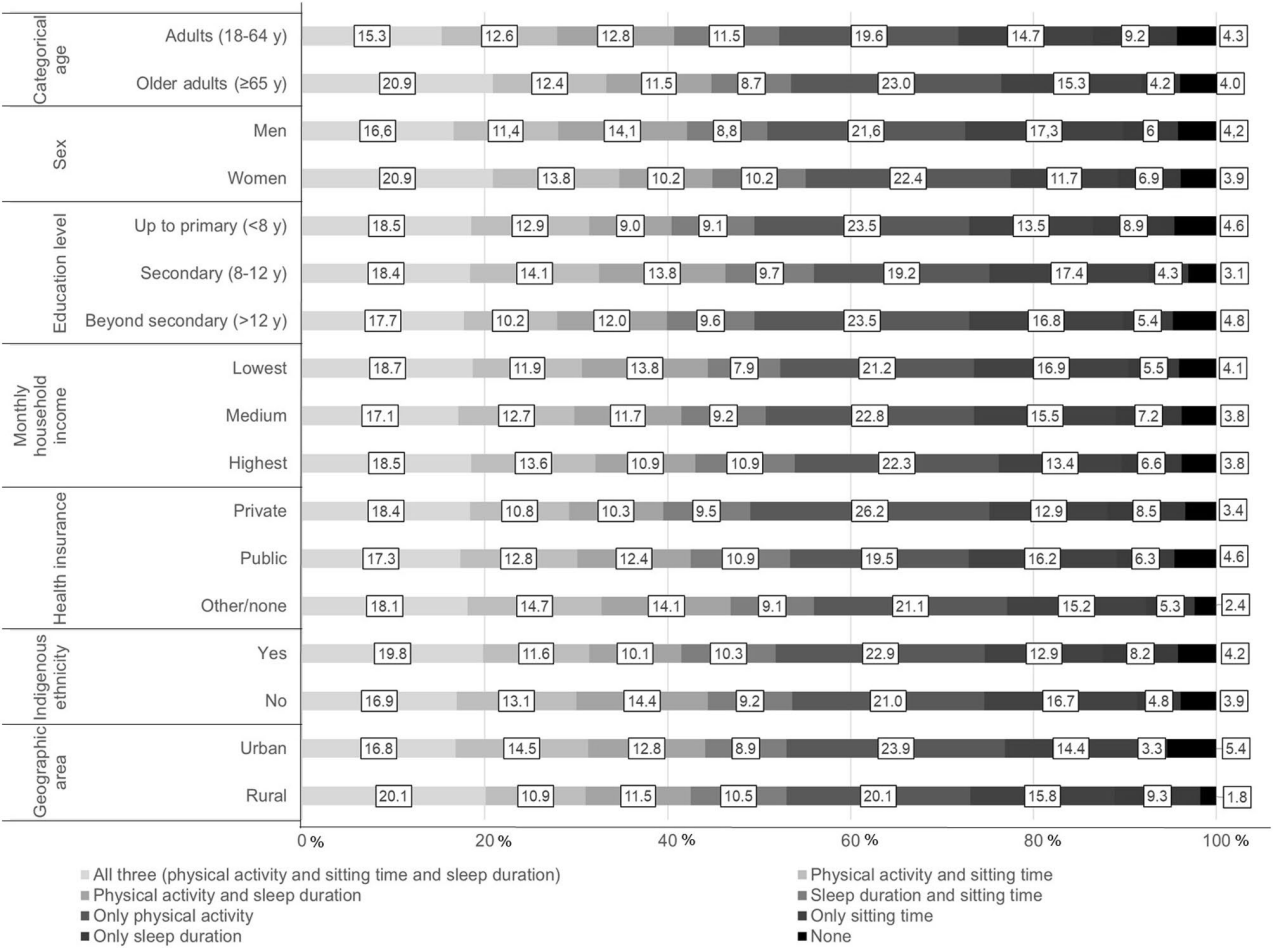
Proportion (%) of participants meeting the specific combinations according sociodemographic characteristics.

Figures 2 and 3 show the proportion of participants meeting the general and specific combinations according sociodemographic characteristics. A total of 16.6% of men and 20.9% of women met all three recommendations, whereas 4.2% and 3.9% met none of the three recommendations, respectively. Older adults, women, those with up to primary education level, indigenous ethnicitiy and living in rural areas were more likely to meet all three 24-h movement guidelines.

Not meeting any 24-h movement guidelines was associated with higher odds of body mass index (OR 1.67; 95% CI 1.45 to 1.89), waist circumference (OR 1.65; 95% CI 1.40 to 1.90), hypertension (OR 2.88; 95% CI 2.23 to 3.53), type 2 diabetes (OR 1.60; 95% CI 1.26 to 1.94), metabolic syndrome (OR 1.97; 95% CI 1.54 to 2.40) and risk of cardiovascular disease (OR 1.50; 95% CI 1.20 to 1.80) compared to participants meeting all three 24-h movement guidelines. Compared to participants meeting all three 24-h movement guidelines, those meeting only one out of three guidelines had higher odds of triglycerides (OR 1.75; 95% CI 1.40 to 2.10), hypertension (OR 1.44; 95% CI 1.11 to 1.77), type 2 diabetes (OR 2.61; 95% CI 2.10 to 3.12), metabolic syndrome (OR 1.54; 95% CI 1.11 to 1.97), and risk of cardiovascular disease (OR 1.12; 95% CI 1.05 to 1.19). These associations were not statistically significant when compared participants meeting two vs all three guidelines (Table 3).

**Table 3:** Multivariable logistic regression models for the associations of meeting physical activity, sitting time, sleep duration, and combinations of these recommendations with cardiometabolic health in Chilean adults.

Meeting guidelines	BMI (overweight/obesity) OR (95%CI)^a^	WC (above threshold) OR (95%CI)^b^	Triglycerides (yes) OR (95%CI)^c^	Hypertension (yes) OR (95%CI)^c^	Type 2 diabetes (yes) OR (95%CI)^c^	Metabolic syndrome (yes) OR (95%CI)^c^	Risk of CVD (middle/high) OR (95%CI)^d^
Guidelines met all three	1.00 (Reference)	1.00 (Reference)	1.00 (Reference)	1.00 (Reference)	1.00 (Reference)	1.00 (Reference)	1.00 (Reference)
Guidelines met two out of three	1.15 (0.90 to 1.40)	0.92 (0.72 to 1.12)	1.08 (0.88 to 1.28)	0.81 (0.56 to 1.06)	0.70 (0.33 to 1.07)	1.11 (0.85 to 1.37)	0.72 (0.58 to 0.90)
Guidelines met one out of three	0.80 (0.37 to 1.23)	0.72 (0.35 to 1.09)	1.75 (1.40 to 2.10)*	1.44 (1.11 to 1.77)*	2.61 (2.10 to 3.12)*	1.54 (1.11 to 1.97)*	1.12 (1.05 to 1.19)*
Guidelines met none out of three	1.67 (1.45 to 1.89)*	1.65 (1.40 to 1.90)*	1.21 (0.95 to 1.38)	2.88 (2.23 to 3.53)*	1.60 (1.26 to 1.94)*	1.97 (1.54 to 2.40)*	1.50 (1.20 to 1.80)*
Guidelines met PA and ST	1.10 (0.90 to 1.30)	0.75 (0.15 to 1.35)	1.06 (0.90 to 1.22)	0.91 (0.67 to 1.15)	1.45 (0.91 to 2.36)	1.83 (0. 79 to 2.87)	0.99 (0.83 to 1.19)
Guidelines met PA and sleep duration	1.16 (0.96 to 1.36)	1.02 (0.81 to 1.23)	1.15 (1.06 to 1.24)*	1.75 (1.40 to 2.10)*	1.50 (1.23 to 1.77)*	1.70 (1.26 to 2.14)*	1.15 (0.90 to 1.40)
Guidelines met sleep duration and ST	0.93 (0.27 to 1.59)	1.26 (1.05 to 1.47)*	2.46 (1.46 to 3.46)*	2.02 (1.60 to 2.44)*	2.16 (1.74 to 2.58)*	1.89 (1.44 to 2.34)*	1.67 (0.54 to 2.80)
Guidelines met only PA	1.89 (1.50 to 2.28)*	1.70 (1.36 to 2.04)*	1.10 (0.83 to 1.37)	2.68 (2.15 to 3.21)*	1.47 (1.13 to 1.81)*	1.26 (1.04 to 1.48)*	1.50 (1.21 to 1.79)*
Guidelines met only ST	1.27 (1.05 to 1.49)*	1.08 (0.83 to 1.33)	1.44 (1.12 to 1.76)*	1.90 (1.40 to 2.40)*	1.50 (1.03 to 1.97)*	2.16 (1.34 to 2.98)*	1.30 (1.06 to 1.54)*
Guidelines met only sleep duration	1.08 (0.96 to 1.20)	0.90 (0.75 to 1.05)	1.02 (0.84 to 1.20)	1.23 (0.97 to 1.49)	1.78 (0.70 to 2.86)	1.12 (0.62 to 1.74)	1.03 (0.60 to 1.46)

*BMI* body mass index, *WC* waist circumference, *CVD* cardiovascular disease, *OR* odds ratio, *95% CI* confidence interval 95%, *PA* physical activity, *ST* sitting time.

Multinomial logistic regression model with cardiometabolic health as dependent variable adjusted for region, sex, age, education level, monthly household income, health insurance, ethnicity, geographic area, fruits and vegetables consumption, tobacco consumption, and alcohol consumption.

^a^0 = underweight/eutrophic; 1 = overweight/obesity.

^b^0 = below threshold; 1 = above threshold.

^c^0 = no; 1 = yes.

^d^0 = low; 1 = middle/high.

*p < 0.05.

In the specific combinations of movement behaviors, meeting both physical activity and sleep duration was associated with higher odds of triglycerides (OR 1.15; 95% CI 1.06 to 1.24), hypertension (OR 1.75; 95% CI 1.40 to 2.10), type 2 diabetes (OR 1.50; 95% CI 1.23 to 1.77), and metabolic syndrome (OR 1.70; 95% CI 1.26 to 2.14) compared to met all three meeting 24-h movement guidelines. Furthermore, sleep duration and sitting time was associated with higher odds of five (waist circumference, triglycerides, hypertension, type 2 diabetes, and metabolic syndrome) of out seven cardiometabolic indicators compared to meeting all three recommendations. Meeting a combination of physical activity and sitting time vs meeting all three guidelines was not associated with a higher odds of all cardiometabolic health indicators (Table 3).

Meeting only the physical activity or only sitting time guidelines were associated with higher odds of six (except triglycecerides for physical activity, and waist circumference for sitting time) out of seven cardiometabolic indicators compared to meeting all three 24-h movement guidelines. Meeting only sleep duration guidelines vs meeting all three guidelines was not associated with a higher odds of all cardiometabolic health indicators (Table 3).

## Discussion

Using a representative sample of adults from Chile, our study examined the associations between different combinations of meeting 24-h movement guidelines and cardiometabolic health in Chilean adults. We found that 18% of participants met all three 24-h movement guidelines. Participants meeting none out of three 24-h movement guidelines had higher odds of overweight/obesity, above threshold waist circumference, hypertension, type 2 diabetes, metabolic syndrome and risk of cardiovascular disease compared to meeting all three guidelines. The results also showed that meeting only one of the three 24-h movement guidelines was associated with higher odds of having several cardiometabolic risk factors compared to meeting all three guidelines.

This study is the first to examine the prevalence of 24-h movement guidelines in Chilean adults. Our study may also contribute to the scientific evidence regarding the association of movement behavior with health outcomes. In line with the findings of our analyses, two recent systematic reviews with compositional data analysis studies suggested that the composition of movement behaviors across the 24-h day (including physical activity, sitting time, and sleep duration) was associated with cardiometabolic health indicators (i.e., body mass index, waist circumference, triglycerides, hypertension)^6,36^.

To properly analyze movement behaviours constrained to, but filling, the 24-h period compositional analyses are recommended^9,17^. As none of the 24-h movement behaviors are independent of each other, and each of these behaviors has reciprocal effects on the others^37^, it has been recommended that it is unsuitable for studying their associations with important health implications separetely. Compositional data analysis methods allow for general 24-h time use among dissimilar movement behaviors to be assessed. Compositional approaches address multicollinearity issues between movement variables, ensure that estimates are fully adjusted for all-time habit, and allow for the inspection of mutual and synergistic associations of the 24-h movement behaviors with health indicators^38^. Our findings support the importance the association between the daily composition of movement behaviors and cardiometabolic health.

This study found that meeting none and one out of three recommendations was associated with a increased odds of cardiometabolic risk compared to meeting all three guidelines. Even specific combinations of meeting 2 out of 3 movement guidelines may not suffice to ensure optimal cardiometabolic health. Rao et al. also found a dose–response relationship between the number of movement guidelines met and physical, mental, and social health outcomes, such as physical activity, screen time, prosocial behaviours, and life satisfaction^39^. Similar results were also reported in children with a dose–response relationship between meeting movement guidelines and reduced risk of obesity^40^. Our study, however, showed that the combination of sufficient physical activity and lower sitting time may provide beneficial effects on maintaining cardiometabolic health. Meeting sleep duration and sitting time, on the other hand, was associated with higher odds of five (waist circumference, triglycerides, hypertension, type 2 diabetes, and metabolic syndrome) out of seven cardiometabolic indicators compared to meeting all three recommendations. Only meeting sleep duration guidelines, however, was not associated with cardiometablic health indicators compared to meeting all three guidelines.

In a recent study that examined the temporal and bidirectional relationship between objectively-measured sleep duration, sitting time, and physical activity, results indicated that higher levels of physical activity were related with adequade sleep duration, whereas increased sitting time was concomitant with poor sleep duration^41^, and these interactions may influence cardiometabolic health indicators. The complex temporal and reciprocal relationships between the 24-h movement behaviors remains poorly understood. Clarifying these interactions, and their relationship with other outcomes is essential for notifying targeted intervention approaches to increase the amount of people meeting all movement guidelines in order to reduce the burden of non-communicable diseases. The quantity of studies using compositional analyses and/or assessing compliance with the 24-h movement guidelines to analyse interactions with well-being is rapidly increasing. These findings are essential for understanding 24-h movement behaviors in Latin American adults and establishing evidence-based interventions for preventing cardiometabolic diseases.

The evidence presented in our study suggests that 18.1% of Chilean adults met the integrated 24-h movement behavior recommendations (i.e., a combination of physical activity, sitting time, and sleep duration recommendations). A recent systematic review also highlights the lack of data on compliance of movement guidelines in aduts and older adults^6^. Future studies are necessary to gain a more thorough understanding of the proportion of individuals who simultaneously achieve all of the movement behavior recommendations and the associations with cardiometabolic health indicators.

This study has several limitations. One limitation of the study is the cross-sectional design which cannot reveal the temporal relationship between 24-h movement behaviors and cardiometabolic health indicators. Further, the NHS and other publicly available national health surveillance data rely on self-reported measures, which are subject to measurement error. The limited sample size of “none" group means that caution must be exercised when interpreting our findings on guidelines met none. In addition, 3615 participants, were excluded from the analyses due to incomplete data, which may have led to selection bias. However, participants excluded due to missing data were similar (p > 0.05) to those included in our study in terms of sex, age group and educational level. The physical activity guidelines were only calculated based on moderate-to-vigorous physical activity without considering muscle-strengthening activities and other light activites. We also used sitting time to estimate total sitting time. This measure may not capture time spent lying down, for example^42^.

There are several strengths of the present study, however, that should be considered. Associations were adjusted for several potential confounders, such as sex, age, education level, monthly household income, health insurance, ethnicity, geographic area and other lifestyle risk factors. We used objective measurements for body mass index, waist circumference, triglycerides, blood samples, and blood pressure. A large sample size also ensured adequate statistical power. Furthermore, currently, there has been limited data on 24-h movement behavior in Latin America; countries need to develop these to allow greater measurement, surveillance and promotion of movement behaviors among adults in this region.

Our findings, illustrate important considerations that must be made about the content of new guidelines released in Chile. Longitudinal and intervention studies are needed to examine a wide range of cardiometabolic health that are potentially associated with the 24-h movement guidelines. Future studies should also apply device-based measures of physical activity (e.g., accelerometers) or combine them with subjective ones (e.g., diaries and questionnaires) to assess movement behaviors in more detail.

## Conclusions

In summary, around one out of five adults met the 24-h movement behavior guidelines in Chile. Meeting none or one out of three 24-h movement behavior guidelines was associated with higher odds of having poor cardiometabolic indicators in Chilean adults. National public health efforts are needed to promote more physical activity, less sitting time, and adequate sleep duration among Chilean residents to increase the proportion of individuals meeting at least 2, and preferably all three, existing 24-h movement recommendations. Future efforts should, therefore, consider novel strategies to simultaneously improve physical activity, sitting time and sleep duration in adults.

## Data Availability

The datasets generated and/or analysed during the current study are available in the database repository of the Epidemiology Department of the Chilean Ministry of Health: http://epi.minsal.cl/bases-de-datos/.
